# Epigastric pain relating to menses can be a symptom of bowel endometriosis

**DOI:** 10.1590/S1516-31802008000400012

**Published:** 2008-07-03

**Authors:** Sergio Podgaec, Manoel Orlando Gonçalves, Sidney Klajner, Mauricio Simões Abrão

**Keywords:** Endometriosis, Intestines, Laparoscopy, Diagnosis, Therapeutics, Endometriose, Intestinos, Laparoscopia, Diagnóstico, Terapêutica

## Abstract

**CONTEXT AND OBJECTIVE:**

Endometriosis is a common affliction that may affect the intestinal tract. The objective of this case report was to describe an unusual clinical presentation of this form of the disease.

**CASE REPORT:**

The patient was a 35-year-old woman with epigastric pain that only occurred during menstruation, who had a history of bladder endometriosis. Endoscopy of the upper digestive tract showed normal results. Transvaginal ultrasound and nuclear magnetic resonance of the pelvis showed a lesion involving the ileocecal junction and appendix, measuring 30 × 22 × 13 mm, that was suggestive of endometriosis. The patient underwent laparoscopic resection of the bowel segment affected by the disease, followed by anastomosis of the ileum and ascending colon for immediate restoration of intestinal transit. Histological analysis confirmed the diagnosis of endometriosis.

**CONCLUSIONS:**

In young women, recurrent epigastric pain should be evaluated with regard to its relationship to menstruation, particularly if there is a history of endometriosis, since this may be a clinical sign that the disease is affecting the intestinal transit.

## RESUMO

**CONTEXTO E OBJETIVO:**

A endometriose é uma doença freqüente que pode acometer o trato intestinal. O objetivo deste relato de caso é descrever forma pouco usual de apresentação clínica.

**RELATO DE CASO:**

Paciente de 35 anos tinha queixa de dor em região epigástrica somente durante o período menstrual e antecedente de endometriose vesical. Endoscopia digestiva alta era normal, e ultra-sonografia transvaginal e ressonância nuclear magnética da pelve demonstravam lesão em transição íleo-ceco-apendicular de 30 × 22 × 13 mm sugestiva de endometriose. A paciente foi submetida a ressecção laparoscópica do segmento intestinal afetado pela doença com imediata reconstrução do trânsito intestinal através da confecção de anastomose íleo-cólon ascendente. A análise histológica confirmou o diagnóstico de endometriose.

**CONCLUSÕES:**

Dor epigástrica recorrente deve ser caracterizada quanto à sua relação com o período menstrual em mulheres jovens, especialmente se houver antecedente de endometriose, pois pode ser um sinal clínico da doença acometendo o trato intestinal.

## INTRODUCTION

Endometriosis is defined as the presence of ectopic endometrial tissue in extrauterine sites. It affects between 10 and 15% of women of reproductive age and its etiopathogenic mechanisms probably involve immunological abnormalities.^1^ In 1997, it was suggested that endometriosis may be manifested as superficial implants in the pelvic peritoneum, in the form of ovarian chocolate cysts referred to as endometriomas, and/or deeply infiltrated (to a depth of more than 5 mm) in the bladder, ureters, retrocervical region of the uterus, rectovaginal septum and bowel.^2^ This differentiation was a landmark in the therapeutic management of the disease because it led to the perception that its deep infiltrative form should be considered to be a severe type of endometriosis requiring extremely specialized treatment to achieve optimal clinical resolution.

The bowel is involved in approximately 10% of endometriosis cases and, in 90% of these cases, the rectum and/or sigmoid are the portions affected. Other segments are affected more rarely: the appendix, ileum and cecum in 7% of cases and the jejunum and small intestine in 3% of cases.^3^ In these cases, the clinical status is related to pain in the pelvic region, deep dyspareunia and changes in bowel habits such as bleeding and pain on evacuation, particularly during menstruation.

Improvements in imaging methods such as transvaginal ultrasound and MRI and evolution in laparoscopic surgery have enabled greater access to diagnoses of endometriosis. Many questions concerning the clinical and basic scientific aspects of such diagnoses have been included in different fields of research. In this respect, a recent study calculated that the annual cost due to endometriosis reached $22 billion in 2002, which is considerably higher than the costs relating to Crohn's disease or migraine.^4^ The case reported here describes an unusual form of clinical presentation of endometriosis affecting the bowel.

## CASE REPORT

A 35-year-old single white female patient, who was an engineer born in São Paulo, Brazil, presented with a complaint of pain in the epigastric region that only occurred during her menstrual period. It had become worse over the preceding six months to the extent that, in the last two months, she had required hospitalization for endovenous analgesic treatment. The patient reported the pain as colic or pressure in the epigastric region, especially during eating, that did not improve after taking proton pump inhibitors and receded following the end of her menstrual period. The patient reported no episodes of epigastralgia other than during these critical periods.

She reported slight dysmenorrhea that improved with the use of common analgesic medication. She said that she did not have any acyclic pelvic pain (apart from during her menstrual period), dyspareunia or any cyclic urinary or bowel abnormalities (during menstruation). Her menstrual cycles were regular; she had never been pregnant and, at the time of these episodes, she had an active sex life but no wish to become pregnant. She used condoms for contraception. She said that she did not have any concomitant diseases and was not using any medication.

With regard to her medical history, the patient reported that five years previously she had suffered repeated episodes of dysuria, which were also exclusively during menstruation. At that time, she underwent transvaginal ultrasonography and nuclear magnetic resonance, which detected a 3 cm nodule on the wall of her bladder. She then underwent videolaparoscopy for the removal of this nodule, which was affecting the entire length of the bladder wall. Histological analysis revealed that it was a case of endometriosis.

With regard to the complaint of the present report, general physical and gynecological examinations on the patient both showed normal results. During a crisis, only the pain in the epigastric region was evident on abdominal palpation. This pain extended diffusely towards the abdomen, but without any signs of peritoneal irritation.

**Figure 1:**
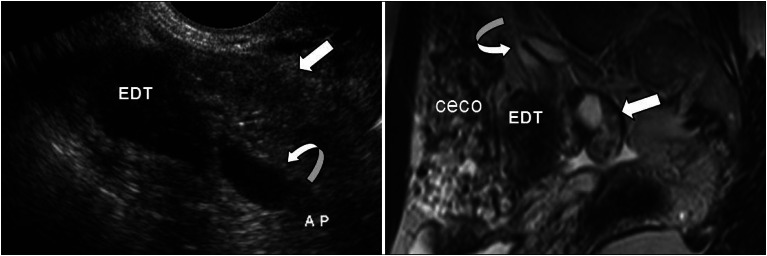
Image of the ileocecal junction and appendix suggestive of endometriosis (EDT), from transvaginal ultrasonography (left) and nuclear magnetic resonance (right). AP = appendix (curved arrow); small bowel shown with the arrow; CECO = ascending colon.

Initially, endoscopy of the upper digestive tract and total abdominal ultrasonography were requested, and these showed normal results. The cyclic nature of the pain and the patient's history of endometriosis led us to request transvaginal ultrasonography with bowel preparation using enema (Fleet^®^) prior to the examination. The results revealed a solid nodular hyperechogenic image involving the ileocecal junction and appendix, measuring 30 × 22 × 13 mm, that was suggestive of endometriosis (Figure 1). Nuclear magnetic resonance was then requested, and this confirmed the suspected diagnosis (Figure 1). Normal results were obtained from colonoscopy.

**Figure 2:**
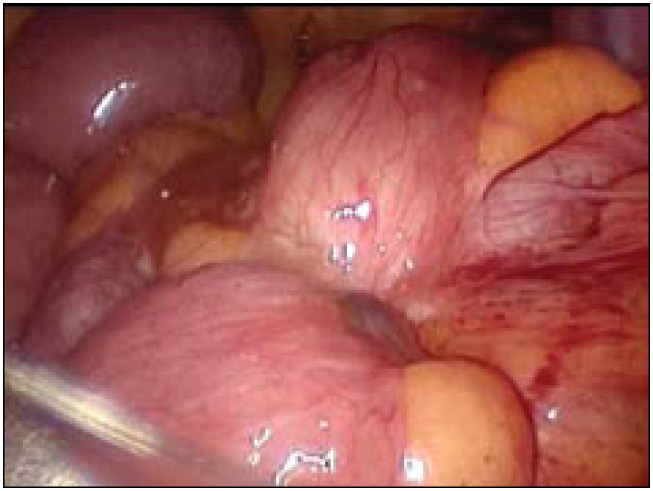
Intraoperative image of lesion at the ileocecal junction compatible with endometriosis.

**Figure 3:**
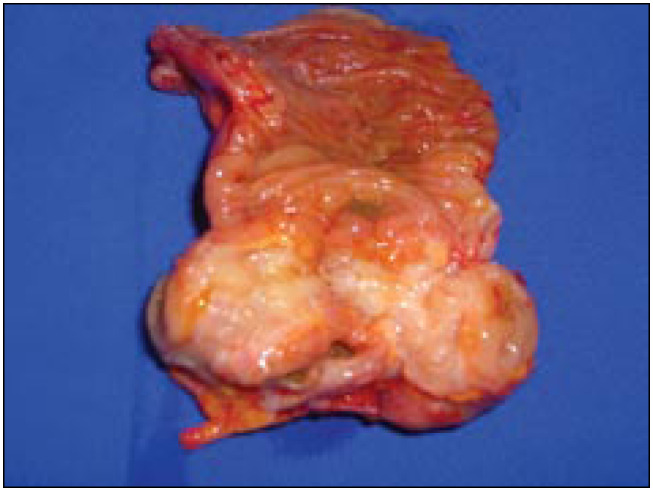
urgical specimen showing lesion at the ileocecal junction compatible with endometriosis.

The patient underwent videolaparoscopy at the Albert Einstein Hospital, São Paulo, Brazil, which revealed the presence of a nodule compatible with the results from the imaging examinations. Laparoscopic resection of the bowel segment affected by the disease was performed (Figures 2 and 3), followed by immediate restoration of the intestinal transit by means of anastomosis of the ileum and ascending colon. Histological analysis confirmed the diagnosis of endometriosis.

Since the patient did not wish to become pregnant immediately, a levonorgestrel-releasing intrauterine device was inserted. There were no postoperative complications. Complete regression of symptoms occurred and, after 12 months of follow-up, the patient remains asymptomatic.

## DISCUSSION

The management of endometriosis has changed radically over recent years, particularly since attention was drawn to the importance of adequately identifying the most aggressive forms of the disease, which are characterized as deep infiltrative endometriosis. The present report describes an unusual situation in which a patient with a history of deep endometriosis had a complaint of intense, cyclic epigastric pain only during her menstrual period, which became progressively worse over a short space of time.

Among the most common symptoms associated with the disease, which include pelvic pain and infertility, epigastric pain is certainly not one of them. One fact that contributed towards the optimal resolution of this situation was that the patient herself, having previously had a nodule of endometriosis in an unusual location (the bladder), perceived that her pain was cyclic and was similar to the dysuria that she had suffered five years previously.

Various cases involving situations of acute bowel obstruction caused by nodules of endometriosis affecting the small intestine have been described, particularly with regard to the terminal ileum.^5,6^ A similar type of clinical situation connecting epigastric pain and endometriosis has already been described in the literature, but in none of those cases was there any characteristic cyclicity relating to the menstrual phase.^7-9^ In the present case, the patient probably had repeated subocclusive episodes during her menstrual period, which led to the epigastric pain that she reported as her principal clinical complaint.

## CONCLUSION

Recurrent epigastric pain should be evaluated with regard to its relationship to menstruation in young women, particularly if there is a history of endometriosis, since this may be a clinical sign that the disease is affecting the intestinal tract.
